# SLC7A11 regulated by NRF2 modulates esophageal squamous cell carcinoma radiosensitivity by inhibiting ferroptosis

**DOI:** 10.1186/s12967-021-03042-7

**Published:** 2021-08-26

**Authors:** Lei Feng, Kaikai Zhao, Liangchao Sun, Xiaoyang Yin, Junpeng Zhang, Conghe Liu, Baosheng Li

**Affiliations:** 1 Shandong First Medical University and Shandong Academy of Medical Sciences, Jinan, 250062 Shandong China; 2 Department of Radiation Oncology, Shandong Cancer Hospital and Institute, Shandong First Medical University, Academy of Medical Sciences, Jinan, 250117 China; 3 Department of Radiation Oncology, Yantai Affiliated Hospital of Binzhou Medical University, Yantai, 264100 Shandong China

**Keywords:** Esophageal squamous cell carcinoma, NRF2, SLC7A11, Ferroptosis, Prognosis

## Abstract

**Background:**

Solute carrier family 7 member 11(SLC7A11) is a component of cysteine/glutamate transporter, which plays a key role in tumor growth; however, its underlying effect on radiosensitivity in esophageal squamous cell carcinoma (ESCC) remains unclear. This study aimed to clarify SLC7A11’s expression and correlation with nuclear expression of nuclear factor erythroid-2 **(**NRF2)-associated radioresistance in ESCC.

**Methods:**

We included 127 ESCC patients who received radical chemoradiotherapy. Immunohistochemical staining was used to detect SLC7A11 and NRF2 nuclear expression, and the relationship between clinicopathological characteristics and survival rates or therapy response were evaluated. Western blot, dual-reporter assays and Chromatin immunoprecipitation (ChIP)-sequencing were used to analyze their relationship in vitro. Their roles in radioresistance were then investigated through multiple validation steps.

**Results:**

NRF2 nuclear expression and SLC7A11 expression were overexpressed in ESCC tissues and were positively correlated with one another. NRF2 nuclear expression was significantly associated with tumor length, lymph node metastasis, and TNM stage, while SLC7A11 expression was associated with lymph node metastasis. Patients with high NRF2 nuclear expression and SLC7A11 expression had significantly shorter overall and progression-free survival, and poor treatment response. The multivariate model showed that NRF2 nuclear expression and SLC7A11 expression, sex and tumor location are independent prognostic factors. In vitro analysis confirmed that hyperactivation of NRF2 induced SLC7A11 expression by directly binding to its promoter region, promoting radioresistance, reducing radiotherapy-induced lipid peroxidation levels, PTGS2 expression, and radiotherapy-related ferroptosis morphologic features.

**Conclusion:**

Our study reveals a connection between high SLC7A11 expression and NRF2 nuclear expression in patients with ESCC that was related to worse survival and poorer therapy outcomes. SLC7A11-mediated ferroptosis inhibition induced NRF2-associated radioresistance, highlighting potential of NRF2/SLC7A11/ferroptosis axis as future therapeutic targets against therapy resistance biomarker.

**Supplementary Information:**

The online version contains supplementary material available at 10.1186/s12967-021-03042-7.

## Background

Chemoradiotherapy (CRT) remains an important treatment strategy in esophageal squamous cell carcinoma (ESCC); however, high heterogeneity with unclear molecular classifications leads to various treatment outcomes [1, 2], and resistance to therapy limits therapy efficacy to a large extent. Therefore, there is an urgent need to identify accurate biomarkers that predict and improve CRT response.

The cystine/glutamate antiporter SLC7A11 (or xCT) imports cystine for glutathione biosynthesis and antioxidant defense and is overexpressed in a variety of human cancers [3]. Recently, SLC7A11 has been found to play an important role in tumor growth, progression, and metastasis in various types of cancer including pancreatic tumors [4], glioblastomas [5], breast cancer [6], esophageal tumors [7], and ovarian cancer [8]. Overexpression of SLC7A11 suppresses ferroptosis [9, 10]—a form of regulated cell death induced by excessive lipid peroxidation—correlating with better radiotherapy response and longer survival in cancer patients [11, 12].

NRF2 is an important transcription factor that regulates cellular antioxidant response [13] and is associated with radioresistance as illustrated in numerous studies [14–16]. However, the exact molecular mechanism of NRF2-mediated radioresistance remains unclear. Normally, low cellular NRF2 concentrations are maintained by proteasomal degradation through a Keap1-Cul3-dependent mechanism [17]. Oxidative stress or similar circumstances, function to stabilize and activate NRF2 in the cell nucleus [18], regulating downstream gene transcription (including SLC7A11); this amplifies glutamate secretion, thereby affecting the tumor microenvironment [19]. NRF2 activation in cancer cells could prevent ferroptosis [13]. Still, whether NRF2-induced radioresistance relates to SLC7A11-mediated ferroptosis regarding ESCC has not been elucidated; additionally, the interaction between hyperactive NRF2 nuclear expression and SLC7A11 on the efficacy of CRT in ESCC patients remains unclear.

We aimed to evaluate NRF2 nuclear expression and SLC7A11 expression using immunohistochemistry (IHC) to determine their prognostic and therapy effects in ESCC patients who received radical CRT. This study is the first to assess the prognostic effects and radiotherapy (RT) response of and SLC7A11 expression in patients with ESCC who underwent radical CRT. Additionally, it provides a potential means of improving ESCC radiosensitivity by targeting the NRF2/SLC7A11/ferroptosis pathway.

## Materials and methods

### Patients

This study was approved by the Ethics Committee of Shandong Cancer Hospital and Institute (approval number: SDTHEC201803); the requirement of informed consent was waived due to the retrospective nature of the study. One hundred twenty-seven patients with ESCC who underwent CRT at our hospital between January 2014 and June 2019 were enrolled in this study. Eligibility criteria were pathology-confirmed ESCC, a Karnofsky performance status score ≥ 70, ≥ 4 chemotherapy (CT) cycles, and an RT dose ≥ 45 Gy. Exclusion criteria were a history of other malignancies, having received RT only, and incomplete records. Patients’ clinicopathological data were collected from their medical records, and the TNM classification was used to determine the tumor stage. Patients were followed up every 3 months posttreatment for the first year, and every 6 months thereafter. The last follow-up time was January 2021.

### IHC staining

Samples obtained via pathological biopsy were routinely fixed in 10% neutral buffered formalin, cut into 3–4 μm sections, and dried for 1 h at 65 °C; sections were deparaffinized with xylene and rehydrated with a graded ethanol series. Antigens were retrieved by heating the samples in EDTA (pH 8.0) for 15–20 min at 95 °C. Endogenous peroxidases were blocked by incubating sections in 3% hydrogen peroxide for 15 min. Protein blocking was performed for 15 min and then incubated with anti-NRF2 (1:250 dilution, ab62352, Abcam, UK) and anti-SLC7A11 (1:200 dilution, ab37185, Abcam) primary antibodies overnight at 4 °C; negative controls were incubated with phosphate buffered solution (PBS) instead of the primary antibodies. On day two, the slides were reheated at room temperature for 1 h, washed 3 times with PBS, 5 min each time, and treated with a Novolink polymer for 10–15 min. Then, appropriate amount of biotin-labelled goat anti-rabbit IgG polymer was dropped, the slides were incubated at room temperature for 10–15 min, washed 3 times with PBS; then appropriate amount of horseradase-labelled streptomycin working solution was dropped for 10–15 min, and washed 3 times with PBS. A working solution was prepared (1:20 DAB chromogen in DAB substrate buffer; Novolink) and applied for 3 min; the slides were then counterstained with haematoxylin (Novolink) for 2 min and dehydrated. All the images were viewed using a BX53 fluorescence microscope (Olympus Corporation, Tokyo, Japan).

### Immunostaining scoring

**Fig. 1:**
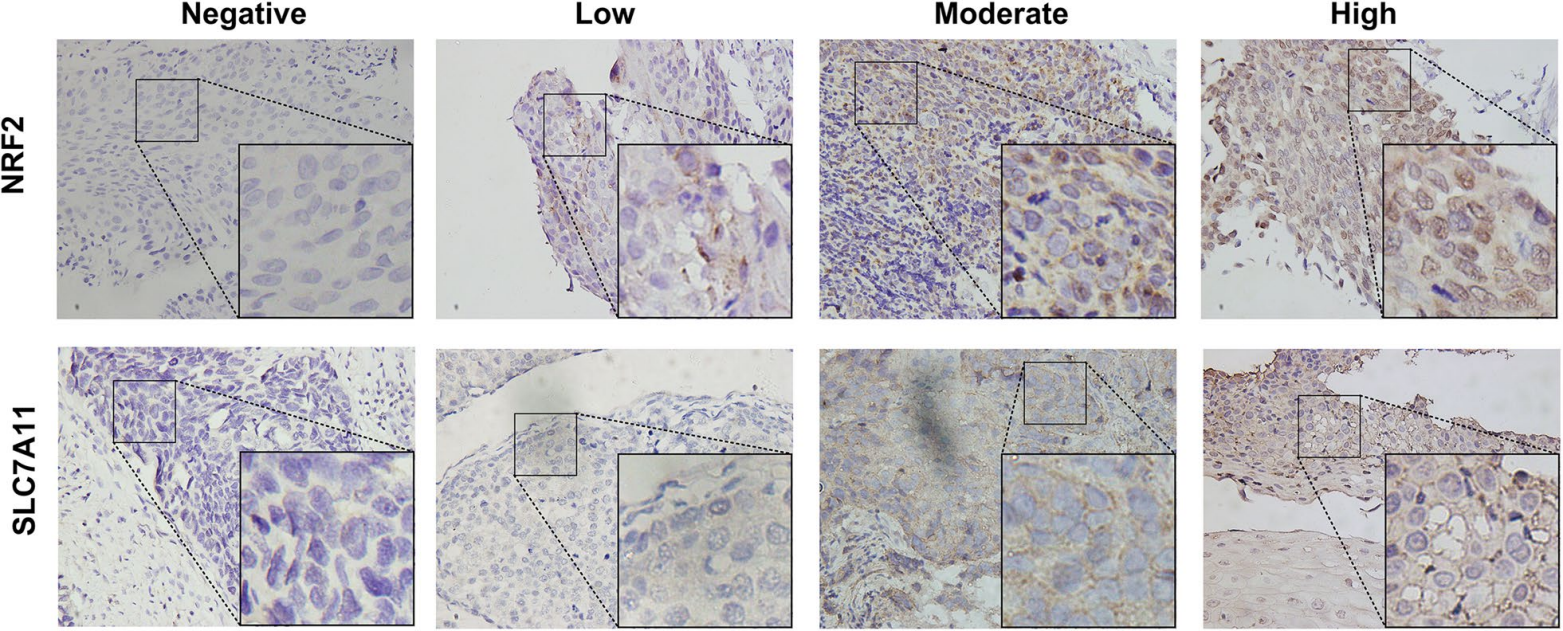
NRF2 and SLC7A11 expression in ESCC tissues. Representative images for negative, low, moderate, and high NRF2 expression; negative, low, moderate, and high SLC7A11 expression (× 400 magnification). ESCC, esophageal squamous cell carcinoma

Five microscopic fields of the tumor tissues (original magnification, ×400) were randomly selected. Two independent investigators, blinded to all immunohistochemical outcomes, adopted a semiquantitative system—considering staining intensity and proportion—to score all the specimens; NRF2 expression was thus semi-quantitatively assessed according to staining intensity in the cancer cells. Reactions were divided into four categories: negative (0, 0%), low (1+, 1–33%), moderate (2+, 34–66%), and high (3+, 67–100%) [20]; expression was observed in either the nucleus or cytoplasm, with an intensity ≥ 1 (Fig. 1). Because NRF2 is an important transcription factor, it usually plays a mechanistic role in the nucleus. Therefore, we focused on the assessment of tumor nuclear expression. Staining of 0 or 1+ was defined as negative, and 2+ or 3+ was defined as positive.

SLC7A11 was mainly expressed on the membrane; it was divided into negative staining (same color as the background), low staining (slightly stronger color than the background), moderate staining (markedly stronger color than the background), and high staining; these were scored as 0, 1, 2, and 3, respectively. According to the percentage of the positive cells in the field, < 10%, 10–25%, 26–75% and > 76% were scored as negative (0), low (1+), moderate (2+), and high (3+) staining, respectively. These two values were subsequently combined to determine the protein expression of each group (a final score of 0–2 represented negative ‘−’ staining, while 3–7 represented positive ‘+’ staining) [21].

### Cell lines and lentivirus infection

The human ESCC cell lines KYSE 30, KYSE 150, KYSE450, KYSE410, KYSE510, and TE-1 were obtained from the Key Laboratory of Shandong Cancer Hospital and Institute. The cells were maintained in DMEM supplemented with 10% FBS and 1% penicillin–streptomycin antibiotics (Gibco Laboratories; Thermo Fisher Scientific, Massachusetts, USA) at 37 °C in a 5% CO_2_ incubator (Galaxy 170R, Eppendorf, Hamburg, Germany). To overexpress NRF2, KYSE 150 and KYSE 30 cells were seeded in six-well plates; once 30–40% confluence was reached, lentiviruses carrying NRF2 (NM_006164.5; GenePharma Co., Ltd, Shanghai, China) were added. After 72 h of transduction, positively transduced cells were selected, using puromycin (NeoFroxx, Einhausen, Germany), for at least 3 days. The SLC7A11 siRNA target sequence was 5′-CTGGAGTTATGCAGCTAAT-3′. For transfection, cells were plated at a density of 1 × 10^5^ cells/well in six-well plates; after 24 h, all siRNA were transfected with siRNA-mate transfection reagent (GenePharma Co., Ltd.), and transfection was confirmed.

### Western blot assay and antibodies

Cells were lysed in lysis buffer (Beyotime Biotechnology, Nanjing, China) containing protease inhibitor cocktails. Nuclear protein extraction and BCA Protein Assay Kits (Beyotime Biotechnology) were used for extraction and to determine protein concentrations, respectively. SDS-PAGE (10%) and Western immunoblotting were performed according to standard procedures; proteins were detected with antibodies recognizing SLC7A11 (1:1000 dilution, ab175186; Abcam), NRF2 (1:500 dilution, ab62352; Abcam) and GAPDH (1:2000 dilution, **#**5174; Cell Signaling Technology, Massachusetts, USA).

### Quantitative-PCR (qPCR)

Total RNA was extracted from the ESCC cell lines using the RNAsimple Total RNA Kit (TIANGEN, Beijing, China). For cDNA synthesis, 500 ng of total RNA was reverse transcribed in 10 μl reaction volume using the PrimeScriptTM RT Master Mix (Perfect Real Time) (Takara Bio Inc., Shiga, Japan). After amplification, the resulting cDNA was subjected to qPCR using TB Green Premix Ex Taq II (Tli RNaseH Plus, Takara Bio Inc.); GAPDH was selected as the housekeeping gene. The expression level of each gene was calculated via the 2^−ΔΔCt^ method. The qPCR primers included: NRF2, 5′-TCCAAGTCCAGAAGCCAAACTGAC-3′ and 5′-GGAGAGGATGCTGCTGAAGGAATC-3′; SLC7A11, 5′-TTACCAGCTTTTGTACGAGTCT-3′ and 5′-GTGAGCTTGCAAAAGGTTAAGA-3′; and GAPDH, 5′-GACCCCTTCATTGACCTCAAC-3′ and 5′-CTTCTCCATGGTGGTGAAGA-3′.

### Cell proliferation assay and clonogenic survival assay

Cell viability was measured with the use of the CCK-8 assay (Bioss, Beijing, China). A 96-well plate was seeded with 2000 cells/well and cultured at 37 °C with 5% CO_2_. The cells were pre-treated with 5 μMFerrostatin-1 (Cat#SML0583, Sigma-Aldrich, Missouri, USA) or DMSO (Beijing Solarbio Science & Technology Co., Ltd., Beijing, China) for 24 h before RT. RT was conducted with a cabinet irradiator (Rad Source Technologies, Georgia, USA) at doses of 6 Gy, and a dose rate of 165 MU/min. Cell viability was determined following the manufacturer’s instructions, and the optical density (OD) value was measured at 450 nm using a microplate reader (SpectraMax; Molecular Devices, California, USA). In addition, the blank background group—wells with only DMEM medium was set up to eliminate the OD value of medium. The calculation formula for the cell proliferation rate is: cell proliferation rate (%) = (OD treatment group−OD Blank)/(OD control group−OD Blank) × 100% [22]. Cell survival following radiation exposure was defined as the cell’s ability to maintain clonogenic capacity and subsequently form colonies. Cells were counted and seeded in six-well plates at 500 cells/well. After 24 h pre-treatment with 5 μM Ferrostatin-1, the cells were exposed to the indicated doses (0–6 Gy) of RT and incubated at 37 °C for 12–14 days. Colonies were stained with crystal violet, manually counted, and those consisting of ≥ 50 cells were scored. All experiments were performed in triplicate.

### Reactive oxygen species (ROS) and lipid peroxidation assay

ROS and lipid peroxidation levels were measured as previously described [23, 24]. Cells were seeded in six-well plates 24 h prior to RT and incubated for another 24 h; 4 μM CM-H2DCFDA (ThermoFisher, C6827, USA) for the total ROS measurements or 5 μM BODIPY 581/591 C11 dye (Invitrogen, D3861, USA) for measurements of the lipid peroxidation levels was added per well, along with fresh medium. After incubation (30 min), cells were washed with PBS, trypsinized to a cell suspension with Trypsin–EDTA solution (Biosharp, Shanghai, China), and analyzed via flow cytometry (FACSCalibur; BD Biosciences, New Jersey, USA).

### Transmission electron microscopy (TEM)

TEM analyses were performed as previously described [24]. Cells were collected by gently scraping and fixing samples with 4% paraformaldehyde (PFA) (Bioss, Beijing, China) for at least 2 h at 4 ℃, and dehydrated, at increasing concentrations of ethanol and propylene oxide after fixing with 0.1% osmium tetroxide. We then embedded the samples by cutting them into ultra-thin sections and staining them with 3% uranyl acetate and lead citrate; these were examined under the microscope (HT-7800; Hitachi Medical Corporation, Tokyo Japan).

### Dual-reporter assays

Transient transfection of 5 × 10^4^ 293T cells with reporter luciferase plasmids and co-transfected plasmids (Shanghai GeneChem Co., Ltd., Shanghai, China) was performed in 24-well culture plates using the Lipofectamine 3000 Transfection Reagent (Invitrogen, Themo Fisher Scientific) as previously described [25]. Cells were harvested 48 h after transfection, and luciferase activities were measured using the Dual-Glo Luciferase Assay System (Promega Corporation, Wisconsin, USA). Transfection and reporter assays were performed in duplicate, and independently repeated at least three times.

### ChIP-sequencing analysis

As were used in previous studies [26], KYSE 150 cells (> 1 × 10^7^) were crosslinked with 1% formaldehyde for 10 min at room temperature and quenched with 0.125 mol/L glycine for 5 min. In brief, nuclei were isolated by dounce homogenization and subjected to sonication to generate 200–1000 bp fragments in length, a portion of it was used to be the input DNA. The Protein-DNA complexes were immunoprecipitated with protein A + G magnetic beads coupled to the anti-NRF2(Cell Signaling Technology, 12721, America). ChIP DNA then were obtained by reverse cross-linking with proteinase K and phenol chloroform. ChIP DNA libraries were created in the following six successive steps: end repair (NEB No. E6050S); dA tailing (NEB No. E6053S); adaptor ligation (NEB No.E7445S); size selection (Beckman, Agencourt AMPure XP); PCR amplification (NEB No. M0544S) using a PCR program (at 98 ℃ for 1 min, 12 cycles of 98 ℃ for 10 s, 62 ℃ for 1 min, 72 ℃ for 1 min); and finally extended to 72 ℃ for 5 min. The constructed libraries were cleaned with Ampure beads and sequenced on Illumina HiSeq Xten system. Raw reads were filtered to obtain high quality clean reads by removing sequencing adapters, short reads (length < 50 bp) and low-quality reads using Cutadapt (v1.9.1) and Trimmomatic23 (v0.35). Next, FastQC was used to ensure high reads quality. The clean reads were mapped to the reference Homo_sapiens_assembly38 (hg38) using the Bowtie2 (v2.2.6) software. Peak detection was performed using the MACS (v2.1.1) peak finding algorithm with 0.01 set as the P value cut-off. Annotation of peak sites to gene features was performed using the ChIPseeker R package.

### Statistical analysis

Clinicopathological features were reported in frequencies. Chi-squared and Fisher's exact tests were used for categorical variables, and survival analysis was performed using the Kaplan–Meier method. Univariate and multivariate Cox regression hazards models were used to evaluate survival risk factors, and variables (*p* < 0.2) from univariate analysis were subject to multivariate analysis. An unpaired two-sided Student’s t-test was used to compare groups at each time point, after ensuring that variances were similar between groups. Data are presented as the mean ± SEM for three or more independent biological replicates. Statistical analyses were conducted using IBM SPSS Statistics for Windows, version 22.0 (IBM, Armonk, New York, USA) and GraphPad Prism 8.0 (GraphPad Software Inc., California, USA); *p* < 0.05 was considered statistically significant.

## Results

### Clinicopathological characteristics

The clinicopathological characteristics of the study cohort (n = 127) are summarized in Table 1. Patient age ranged from 43–84 (median: 66) years; 34 patients received RT alone, 93 received CRT, and 97 received an RT dose > 54 Gy. Patients who received CRT were treated with 4 to 6 courses of cisplatin-based CT (combined with paclitaxel/ docetaxel). Eighty (63.0%) patients were men, 61 (48.0%) patients smoked, and 47 (37%) patients had a history of alcohol abuse. The tumor length ranged from 2.0–12.0 cm (median: 5 cm). The number of patients with TNM stages T1-T4 was 3, 19, 77 and 28, respectively. The number of patients staged N0–3 was 26, 79, 19, 3, respectively, M0–1 was 114 and 13, respectively, and TNM stages I–IV were 1, 30, 59, and 37, respectively.

**Table 1:** The clinical characteristics of 127 ESCC patients

Variables	ESCC, n (%)
Sex	
Male	80 (63.0)
Female	47 (27.0)
Age	
Range (Median)	66 (43–84)
Alcohol abuse	
Yes	47(37)
No	80 (63)
Smoking	
Yes	61 (48)
No	66 (52)
Location of primary tumor	
Upper or middle	56 (44.1)
Lower	71 (55.9)
Length, cm	
Range (Median)	5 (2–12)
T stage	
T1–2	22 (17.3)
T3–4	105 (82.7)
Lymph node metastasis	
N0–1	105 (82.7)
N2–3	22 (17.3)
Metastasis	
M0	114 (89.8)
M1	13 (10.2)
TNM stage	
I–II	31 (24.4)
III–IV	96 (75.6)
Therapy method	
RT	34 (26.8)
CRT	93 (73.2)
RT dose (Gy)	
> 54	97 (76.4)
≤ 54	30 (23.6)

### NRF2 and SLC7A11 overexpression in ESCC tissues

**Fig. 2:**
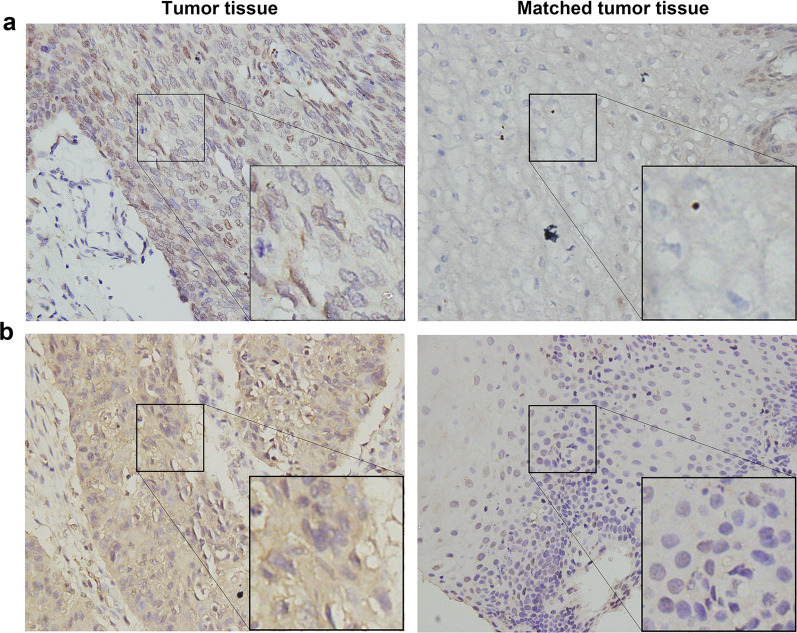
NRF2 nuclear and SLC7A11 expression in ESCC tumor and matched adjacent normal tissues. Representative images of nuclear NRF2 (**a**) and SLC7A11 (**b**) expression in tumor and matched adjacent normal tissues (×400 magnification)

IHC was performed to detect NRF2 nuclear expression (representing NRF2 activation) and SLC7A11 expression. NRF2 and SLC7A11 were positively expressed in 61 (48.0%) and 62 (48.8%) patients, respectively (Fig. 1; Table 3). Additionally, we collected 62 pairs of tumor and matched adjacent normal tissue samples in 62 patients who had undergone radical esophagectomy. NRF2 was positively expressed in 37.1% (23/62) of the tumor samples, compared with 17.7% of the matched adjacent tissue samples (11/62) (*p* = 0.016). Similarly, SLC7A11 expression was higher in tumor tissues than in matched adjacent normal tissues (56.5 versus 14.5%, *p* < 0.001; Table 2; Fig. 2a, b). These results imply that nuclear expression of nuclear NRF2 and SLC7A11 are overexpressed in ESCC tumor tissues.

**Table 2:** Nuclear NRF2 and SLC7A11 expression in ESCC and matched adjacent tissues (n = 62)

Variables	Cancer tissues, n (%)	Matched adjacent tissues, n (%)	P-value
NRF2			0.016
Low or no	39 (62.9)	51 (82.3)	
Moderate or high	23 (37.1)	11 (17.7)	
SLC7A11			< 0.001
Negative	27 (43.5)	53 (85.5)	
Positive	35 (56.5)	9 (14.5)

### NRF2 nuclear and SLC7A11 expression correlates with the clinicopathological characteristics of ESCC

Nuclear expression status of NRF2 and SLC7A11 and the clinical characteristic of patients with ESCC are summarized in Table 3. NRF2 nuclear expression had no significant correlation with age, sex, alcohol abuse and smoking history, tumor location, T stage, or lymph node metastases status. However, it was significantly higher in patients with tumors length ≥ 4 cm (*p* = 0.035) and in patients with later TNM stage (*p* = 0.015). While patients who developed lymph node metastases had significantly higher nuclear expression of NRF2 expression than those with no evidence of metastasis (*p* = 0.037), there was no significant correlation between distant metastasis and nuclear expression of NRF2 (*p* = 0.057).

**Table 3:** Characteristics of patients with ESCC (n = 127)

Variables	NRF2			SLC7A11		
	Low (66)	High (61)	P-value	Negative (65)	Positive (62)	*P-v*alue
Sex			0.189			0.728
Male	38	42		40	40	
Female	28	19		25	22	
Age			0.284			0.872
≤ 60	15	19		17	17	
> 60	51	42		48	45	
Alcohol abuse			0.372			0.698
Yes	22	25		23	24	
No	44	36		42	38	
Smoking			0.803			0.665
Yes	31	30		30	31	
No	35	31		35	31	
Location			0.971			0.632
Upper	29	27		30	26	
Middle/lower	37	34		35	36	
Length, cm			**0.035**			0.623
≤ 4	35	21		27	29	
> 4	31	40		38	33	
T stage			0.790			0.728
T1–2	12	10		12	10	
T3–4	54	51		53	52	
Lymph node metastasis			**0.037**			**0.014**
N0–1	59	46		59	46	
N2–3	7	15		6	16	
Metastasis			0.057			0.333
M0	56	58		60	54	
M1	10	3		5	8	
TNM stage			**0.015**			0.378
I–II	22	9		18	13	
III–IV	44	52		47	49	

**Fig. 3:**
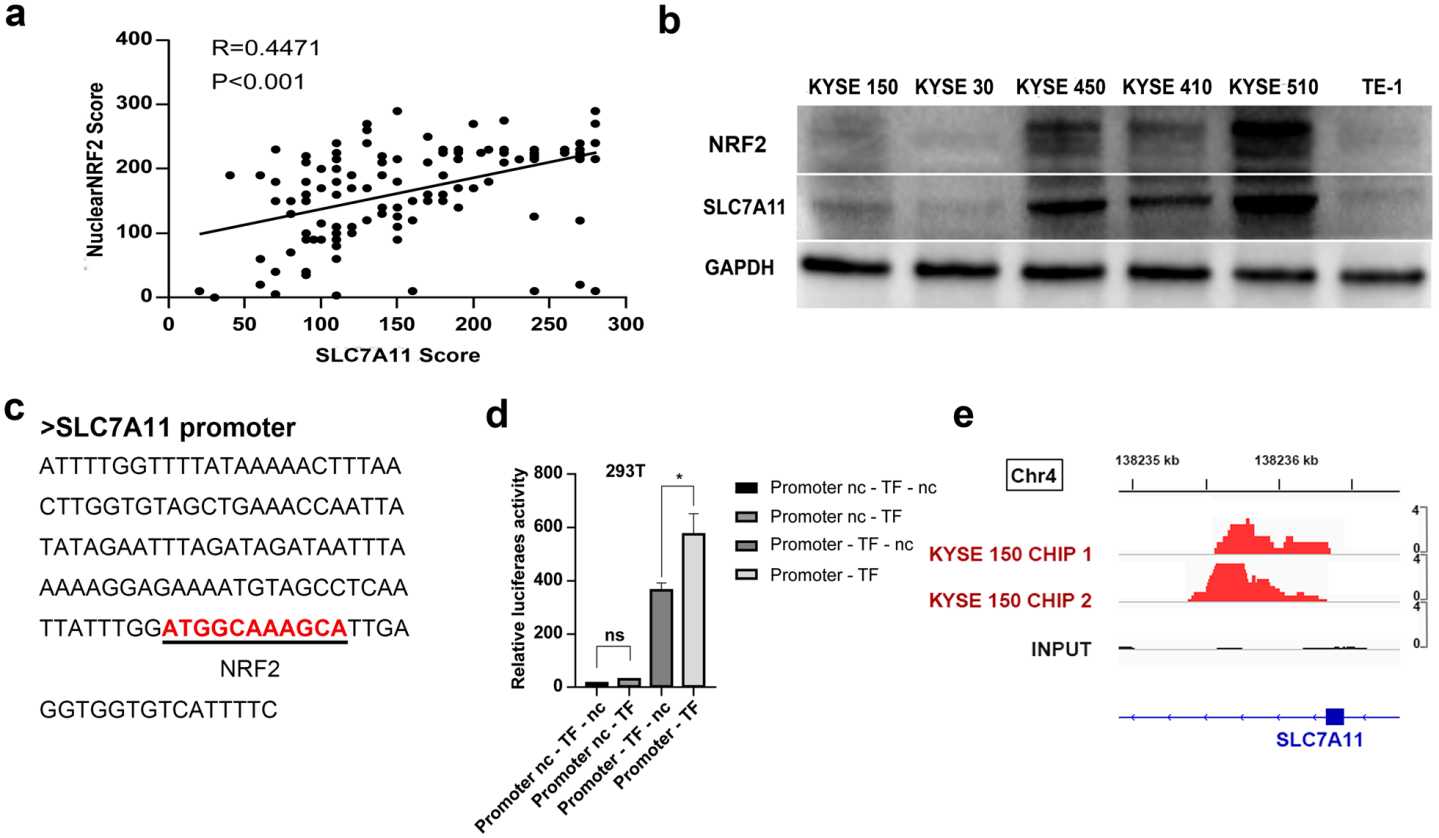
Correlation between SLC7A11 and NRF2 expression. **a** SLC7A11 expression was positively correlated with nuclear NRF2 expression among all the patients (n = 127). **b** SLC7A11 and NRF2 protein expression were analyzed in six ESCC cell lines. Western blotting revealed that SLC7A11 expression positively correlated with NRF2 level. **c** Sequence of the SLC7A11 promoter showing the position of the most representative putative NRF2 binding sites of the promoter. Schematic of putative NRF2 binding sites in the proximal region of SLC7A11 promoter. **d** Dual-luciferase reporter assays showing NRF2 acting as a trans-activator of SLC7A11 promoter activity. Luciferase activity assays (n = 3) were performed after transfection of either firefly luciferase promoter plasmid, NRF2 overexpression plasmid (TF), or TF-nc plasmid into 293T cells. Data are presented as the mean ± SEM. *p < 0.05. **e** Integrative Genomics Viewer (IGV) snapshots of NRF2 binding sites at the promoter of SLC7A11 region

The proportion of SLC7A11-positive patients tended towards high lymph node metastasis rates. Other covariates (age, sex, alcohol, smoking, location, tumor length, and TNM stage) were not significantly associated with SLC7A11 expression, while nuclear expression of NRF2 positively correlated with SLC7A11 (*p* < 0.001, Fig. 3a). These results indicate that NRF2 nuclear expression and SLC7A11 expression are associated with tumor progression and metastasis in ESCC. As we mentioned earlier, the entry of NRF2 into the nucleus activates the NRF2 pathway while escaping ubiquitination and degradation. To validate the relationship between NRF2 and SLC7A11, we conducted further explorations in vitro. Just as we expected, western immunoblotting analysis revealed that NRF2, expressed in six ESCC cell lines, positively correlated with SLC7A11 expression levels (Fig. 3b). Then, we determined whether there is a binding region between transcription factor NRF2 and SLC7A11 promoter using the JASPAR database [27] (http://jaspar.genereg.net/). Strikingly, the results suggested that SLC7A11 was a potential target of NRF2 (Fig. 3c). The dual-luciferase reporter assay showed that hyperactive NRF2 induced SLC7A11 expression by directly binding to its promoter region (*p* = 0.017, Fig. 3d). Moreover, ChIP-sequencing analysis on two highly correlated biological replicates of KYSE 150 identified the recruitment of NRF2 to the SLC7A11 promoter. Strikingly, a strong enrichment of NRF2 signals was detected within the promoter regions of SLC7A11 which is located at chromosome 4, indicating that there is an interaction between NRF2 and SLC7A11 (Fig. 3e).

### Relationship between SLC7A11 or NRF2 nuclear expression and clinical response CRT

**Fig. 4:**
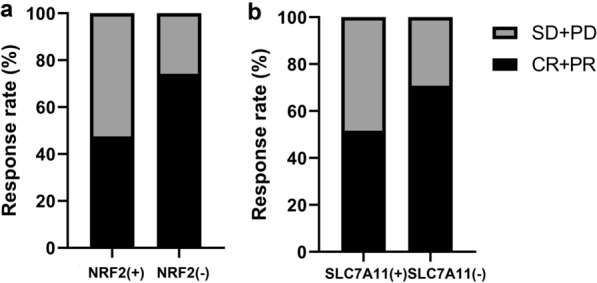
Relationship between SLC7A11 or NRF2 nuclear expression and clinical response to CRT. SLC7A11 (**a**) and nuclear NRF2 (**b**) treatment responses of ESCC patients. CR: complete response; PR: partial response; SD: stable disease; PD: progressive disease

Eleven patients (8.7%) achieved a complete response, 67 patients achieved a partial response, 30 patients (23.6%) experienced stable disease, and 19 patients experienced progressive disease (Fig. 4a, b.). The treatment response was poorer in patients with high NRF2 nuclear expression than in patients with low NRF2 nuclear expression (47.5% vs 74.2%, *p* = 0.002). Similarly, patients with high expression of SLC7A11 had a poorer treatment response than patients with low SLC7A11 expression (51.6% vs 70.8%, *p* = 0.027; Table 4).

**Table 4:** Relationship between SLC7A11 or nuclear NRF2 expression and clinical response to CRT

		Treatment response		
		CR + PR	SD + PD	P-value
Nuclear NRF2	Low expression (%)	49 (74.2)	17 (25.8)	0.002
	High expression (%)	29 (47.5)	32 (52.5)	
SLC7A11	Low expression (%)	46 (70.8)	19 (29.2)	0.027
	High expression (%)	32 (51.6)	30 (48.4)	

### Effect of the NRF2 and SLC7A11 expression on overall survival (OS) and progression-free survival (PFS) in ESCC

**Fig. 5:**
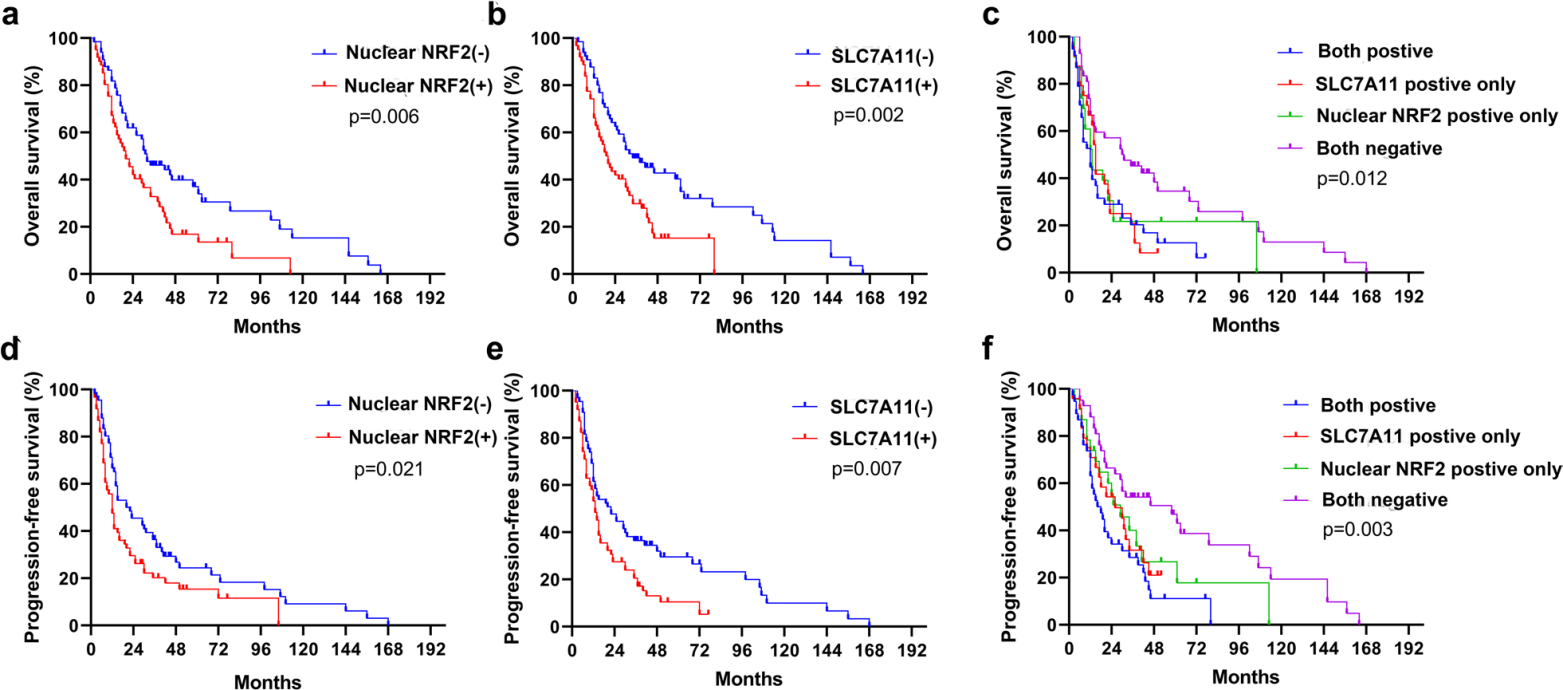
Kaplan–Meier survival curves for NRF2 nuclear expression and SLC7A11 expression. OS survival curves for nuclear NRF2 (**a**), SLC7A11 (**b**), and their combined (**c**) expression. PFS survival curves for nuclear NRF2 (**d**), SLC7A11 (**e**), and their combined (**f**) expression

The NRF2-positive patients had a shorter OS (20 vs 32 months, *p* = 0.006; Fig. 5a) and PFS (12 vs 20 months, *p* = 0.021; Fig. 5d) than the NRF2-negative group. Similarly, SC7A11-positive patients had a shorter OS (19.5 vs 34 months, *p* = 0.002; Fig. 5b) and PFS (13 vs 22 months, *p* = 0.007; Fig. 5e) than SLC7A11-negative patients. Patients negative for both NRF2 and SLC7A11 had the longest OS (mean OS; 30.5 months), whereas double positive expression was linked to the shortest OS (mean OS: 12 months); patients positive for either nuclear expression of NRF2 or SLC7A11 had a similar OS (13 vs 15 months, *p* = 0.012; Fig. 5c). Additionally, patients positive for both NRF2 and SLC7A11 had a shorter mean PFS than those negative for both (17 vs 58 months, *p* = 0.003; Fig. 5f), while patients that were positive for either nuclear expression of NRF2 or SLC7A11 had similar PFS (29 vs 26 months, *p* = 0.741; Fig. 5f). These findings suggest that analyzing NRF2 nuclear expression and SLC7A11 expression status may predict ESCC prognosis received CRT.

### Prognostic value in ESCC

Next, univariate and multivariate Cox regression analysis were used to assess possible prognostic factors in patients with ESCC (Table 5). Univariate analysis revealed that NRF2 nuclear expression and SLC7A11 expression, sex, tumor location, tumor length, T stage, lymph node and distant metastasis, and TNM stage were all associated with OS; conversely, age, alcohol and smoking history were not. Subsequent multivariate Cox regression analysis revealed that sex, tumor location, and NRF2 nuclear expression and SLC7A11 expression were independent prognostic factors for OS.

**Table 5:** Univariate and multivariate analyses of prognostic markers for overall survival in ESCC

Variable	Univariate analysis			Multivariate analysis		
	HR	95% CI	P-value	HR	95% CI	P-value
Nuclear NRF2 expression			0.005			**0.002**
High vs. low or no	1.802	1.191–2.729		1.908	1.256–2.899	
SLC7A11 expression			0.004			**0.010**
Positive vs. negative	1.858	1.220–2.829		1.767	1.146–2.725	
Sex			0.019			**0.022**
Male vs. Female	0.598	0.389–0.919		0.6	0.389–0.927	
Age (years)			0.067			
≤ 60 vs. > 60	1.583	0.969–2.584				
Alcohol abuse			0.200			
Yes vs. No	1.318	0.864–2.011				
Smoking			0.444			
Yes vs. No	1.172	0.781–1.758				
Location			0.002			**0.002**
Upper vs. Middle/Lower	1.898	1.268–2.839		1.908	1.256–2.899	
Length (cm)			0.038			
≤ 4 vs. > 4	1.566	1.025–2.394				
T stage			0.007			
T1–2 vs. T3–4	1.542	1.126–2.112				
Lymph node metastasis			0.001			
N0–1 vs. N2–3	1.741	1.243–2.437				
Distant metastasis			0.004			
M0 vs. M1	2.404	1.321–4.375				
TNM stage			0.001			
I–II vs. III–IV	1.634	1.220–2.188				
Treatment strategy			0.606			
CRT vs. RT	0.888	0.566–1.394				

HR: Hazard ratio; CI: confidence interval

*p < 0.05

### SLC7A11 overexpression induced by hyperactive NRF2 promotes radioresistance by inhibiting ferroptosis

**Fig. 6:**
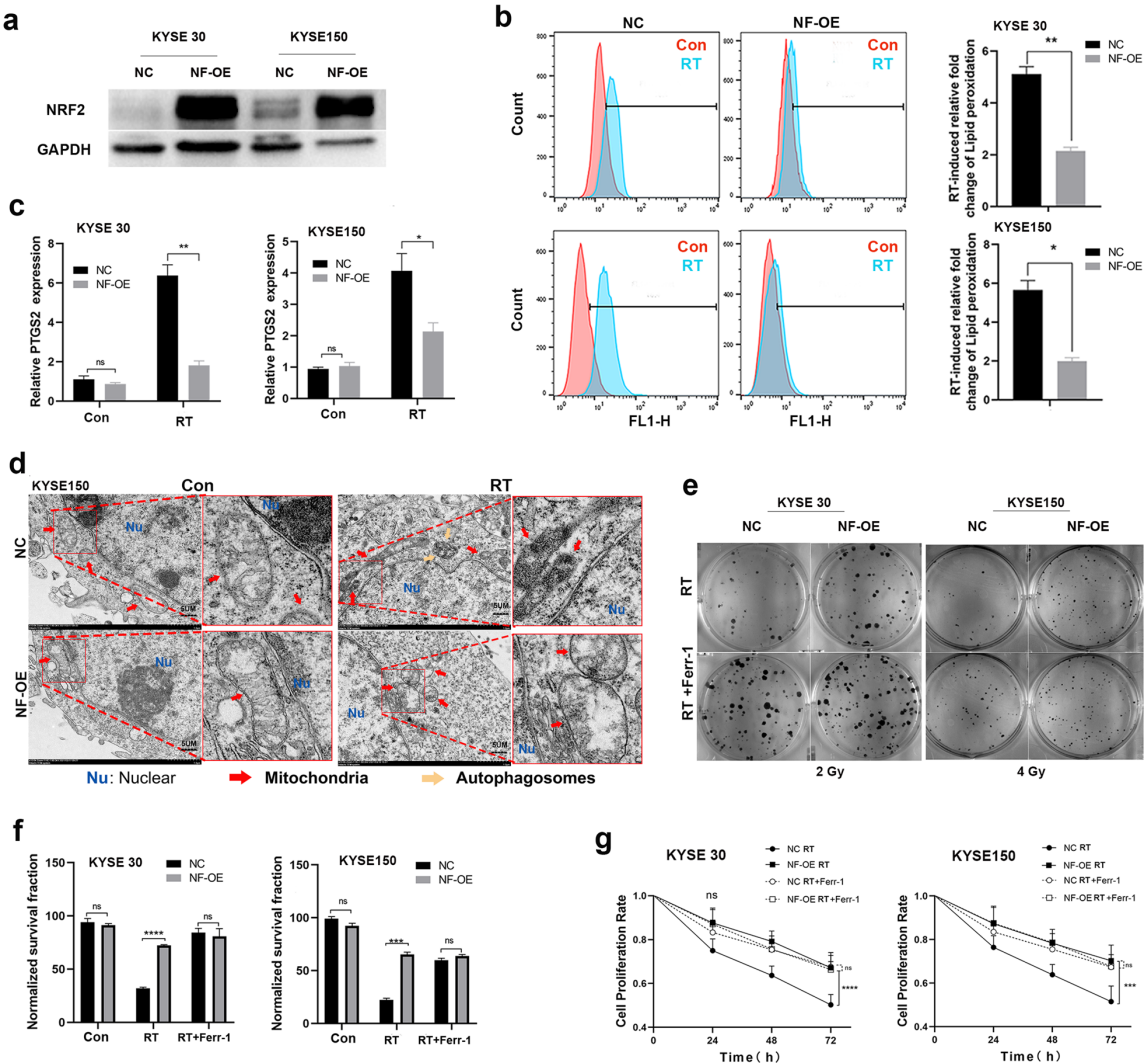
Hyperactive NRF2 promotes radioresistance by inhibiting ferroptosis in ESCC cells. **a** Western blot analysis showing NRF2 levels in KYSE 30 and KYSE 150 cell lines with overexpression of NRF2 (NF-OE), and negative control (NC). **b** Lipid peroxidation assessment of NC and NF-OE 24 h after 6 Gy RT in KYSE 30 and KYSE 150 cells, respectively. Bar graph showing RT-induced relative fold changes of lipid peroxidation levels per 10,000 cells by C11-BODIPY staining in the indicated cells. **c** Quantitative RT-PCR analysis of PTGS2 expression in NC and NF-OE KYSE 30 or KYSE 150 cells 24 h after 6 Gy RT, respectively. **d** TEM images of NC and NF-OE KYSE 150 cells 24 h after exposure to 6 Gy of RT. Nu: nucleus; red arrows: mitochondria; yellow arrows: autophagosome; Scale bars: 5 µm. **e** Representative images of clonogenic survival assays in NC and NF-OE KYSE 30 or KYSE 150 cells pre-treated with 5 μM ferrostatin-1 or DMSO for 24 h, followed by exposure to 2 Gy or 4 Gy of RT, respectively. **f** The quantified clonogenic survival assay in NC and NF-OE KYSE 30 or KYSE 150 cells that were pre-treated with 5 μM ferrostatin-1 or DMSO for 24 h followed by 2 Gy or 4 Gy of RT. **g** Quantified cell proliferation assay curves analyzed by CCK-8 in NC and NF-OE KYSE 30 or KYSE 150 cells pre-treated with 5 μM ferrostatin-1 or DMSO for 24 h, followed by exposure to 6 Gy RT. Each experiment was conducted independently triplicate. Student’s t-test (two-tailed) was used for statistical analysis, *p < 0.05, **p < 0.01, ***p < 0.001, ****p < 0.0001. Bar graphs: mean ± SEM, n = 3

We found a strong correlation between nuclear expression of NRF2 (indicating NRF2 activation) and SLC7A11expression, relating to poor CRT response and shorter OS and PFS in ESCC patients. We reasoned that hyperactive NRF2 promotes radioresistance partly through SLC7A11-mediated ferroptosis inhibition in ESCC. Therefore, we examined whether stabilized NRF2 expression would promote radioresistance by inhibiting ferroptosis in ESCC cell lines. We constructed NRF2 overexpression (NF-OE) in KYSE 30 and KYSE 150 cell lines (Fig. 6a). Considering that NRF2 is an important regulator of the cellular ROS levels [28, 29] and the accumulation of ROS is the trigger for ferroptosis [30, 31], we validated cellular ROS levels and confirmed that (via DCFDA staining, details in Methods and Materials) NRF2 overexpression alleviated the total ROS levels induced by RT in ESCC cells (Additional file 1: Figure S1a). In addition, we found that NRF2 overexpression significantly reduced RT-induced lipid peroxidation levels (analyzed by C11-BODIPY staining), as well as ferroptosis marker gene (PTGS2) expression (Fig. 6b, c) [32]. TEM revealed that RT-treated KYSE 150 (Fig. 6d) and KYSE 30 (Additional file 1: Figure S1b) cells exhibited shrunken mitochondria, increased membrane density and ruptured outer mitochondrial membranes [30]. These are significant ferroptosis-related morphological features [10], including autophagy, which under the exposure of radiation, has been proved to be associated with ferroptosis [33] (such as autophagosomes; Fig. 6d). These phenomena were all largely improved in NRF2 overexpression cells after RT treatment. Consistent with prior literature [34, 35], the data indicated that NRF2 overexpression could promote radioresistance (Fig. 6e–g; Additional file 1: Figure S1c, d).

**Fig. 7:**
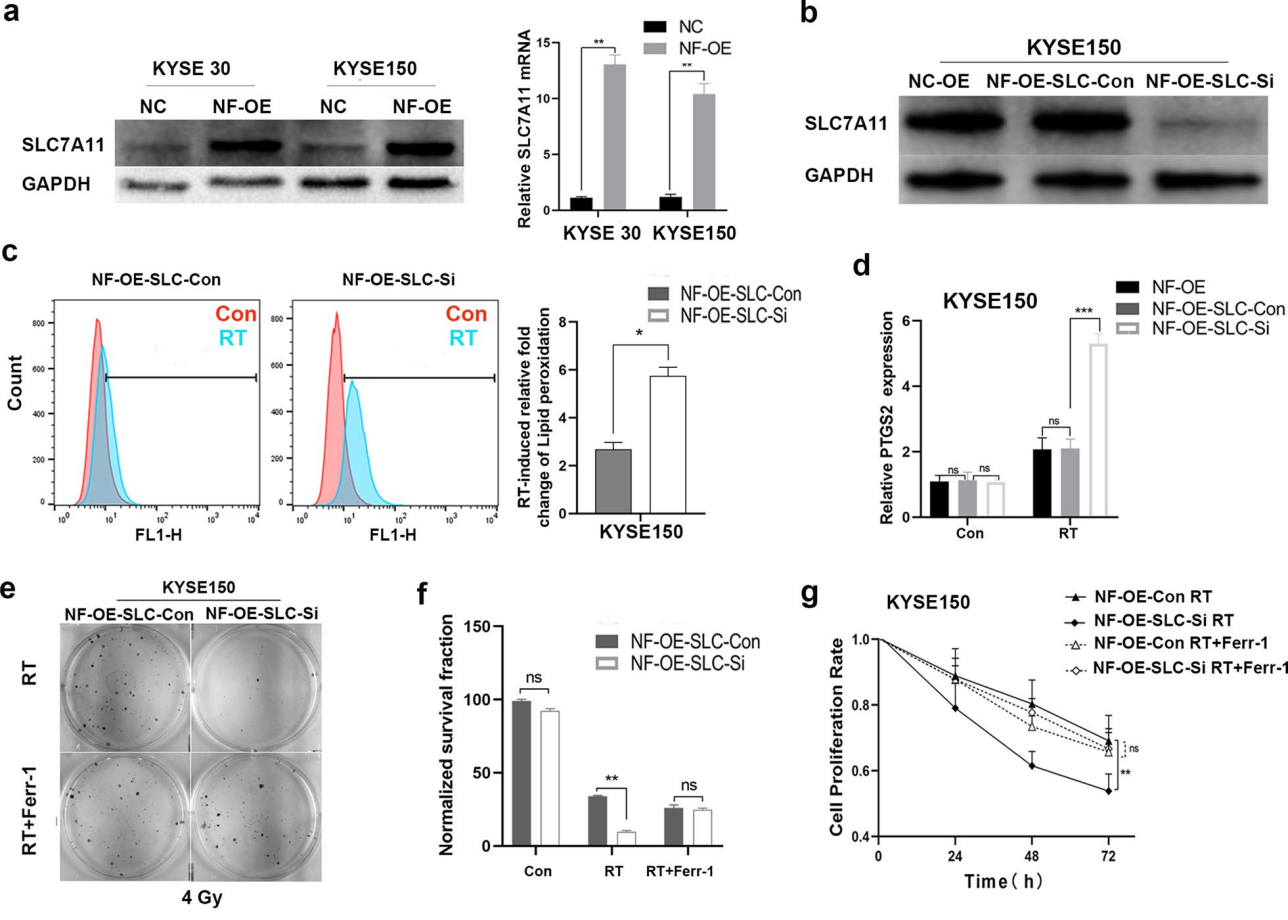
Hyperactive NRF2 inhibits RT-induced ferroptosis by regulating SLC7A11 in ESCC cells. **a** Western blotting and qRT-PCR analysis of NRF2 and SLC7A11 expression in NC and NF-OE KYSE 30 and KYSE 150 cells. **b** Western blot analysis of SLC7A11 levels in NF-OE KYSE 150 cells with SLC7A11 siRNA (NF-OE-SLC-Si), negative control (NF-OE-SLC-con), and NF-OE KYSE 150. **c** Lipid peroxidation assessment in NF-OE-SLC-Si and NF-OE-SLC-con KYSE 150 cells 24 h after exposure to 6 Gy of RT. Bar graph showing RT-induced relative fold changes of lipid peroxidation levels per 10,000 cells by C11-BODIPY staining in the indicated cells. **d** qRT-PCR analysis of PTGS2 expression in NF-OE-SLC-Si and NF-OE-SLC-con KYSE 150 cells 24 h after exposure to 6 Gy of RT. **e** Representative images of clonogenic survival assays in NF-OE-Con and NF-OE-SLC-Si KYSE 150 cells pre-treated with 5 μM ferrostatin-1 or DMSO for 24 h, followed by exposure to 4 Gy of RT, respectively. **f** The quantified clonogenic survival assay in NF-OE-Con and NF-OE-SLC-Si KYSE 150 cells that were pre-treated with 5 μM ferrostatin-1 or DMSO for 24 h followed by 4 Gy of RT, respectively. **g** Quantified cell proliferation assay curves analyzed by CCK-8 in NF-OE-SLC-con and NF-OE-SLC-Si KYSE 150 cells pre-treated with 5 μM ferrostatin-1 or DMSO for 24 h, followed by exposure to 6 Gy RT. Each experiment was conducted independently triplicate. Student’s t-test (two-tailed) was used for statistical analysis, *p < 0.05, **p < 0.01, ***p < 0.001, ****p < 0.0001. Bar graphs: mean ± SEM, n = 3

Notably, the effect of NRF2 overexpression on radioresistance was abrogated under ferroptosis inhibitor ferrostatin-1 treatment (Fig. 6e–g; Additional file 1: Figure S1c, d), suggesting that NRF2 nuclear expression may promote radioresistance by inhibiting ferroptosis; additionally, we found that NRF2 overexpression substantially increased SLC7A11 expression in ESCC cell lines (Fig. 7a). We then knocked down SLC7A11 with siRNA in NRF2 overexpression cells (NF-OE-SLC-Si) to test our hypothesis (Fig. 7b). SLC7A11 deletion in NF-OE KYSE 150 cell lines restored the cellular total ROS levels (Additional file 2: Figure S2a), lipid peroxidation levels and PTGS2 expression reduction induced by RT (Fig. 7c, d), and RT sensitivity was regained (Fig. 7e–g; Additional file 2: Figure S2b, c). Similarly, the effect of SLC7A11 deficiency was abolished with ferrostatin-1 treatment with RT (Fig. 7e–g; Additional file 2: Figure S2b, c). These results suggest that NRF2 overexpression in ESCC cells partly promotes radioresistance through SLC7A11-mediated ferroptosis inhibition.

## Discussion

Numerous studies have recently reported that the hyperactivation of NRF2 contributes to radioresistance in ESCC [14, 16, 36]; however, the underlying mechanism of NRF2-mediated radioresistance remains unclear. Furthermore, accumulating evidence has demonstrated that SLC7A11 promotes therapy resistance partly by suppressing ferroptosis, a form of programmed cell death, that is, induced by excessive lipid peroxidation [10, 12, 37, 38]. In this study, we examined the expression of SLC7A11 and NRF2 in patients with ESCC who received CRT and evaluated their association with clinicopathological characteristics, long-term prognosis, and therapeutic efficacy. Interestingly, the results of clinical analyses revealed a positive correlation between the expression of NRF2 and SLC7A11, indicating a worsening of therapy response and poor prognosis. Emerging evidence demonstrates that the NRF2 signaling pathway plays a significant role in mediating ferroptosis [39–41] and the coeffect between NRF2 and SLC7A11 [42]. We propose that NRF2-associated radioresistance is largely induced by the SLC7A11-mediated inhibition of ferroptosis. To validate this notion, we performed further in vitro validation studies, which are crucial for highlighting the potential of the NRF2/SLC7A11/ferroptosis axis as a possible therapeutic target against biomarkers of therapy resistance.

The nuclear transcription factor, NRF2, is an essential regulator of antioxidant response elements, and the activation of NRF2 signaling plays an important role in protecting cells and tissues from oxidative stress-induced injury [43]. Increasing evidences have demonstrated that NRF2 promotes radioresistance in ESCC [16, 36, 44]; however, the precise mechanistic evidence remains to be elucidated. It has been additionally reported that NRF2 functions as a driver gene for promoting the progression of cancer and metastasis in numerous tumor types, including lung cancer, breast carcinoma cells, and ESCC [13, 45, 46]. However, Li et al. suggested that NRF2 could act as a tumor suppressor in ESCC [2]. Here, the results of our clinical analyses revealed that the overexpression of NRF2 was associated with poor therapeutic outcomes and prognosis in patients with ESCC who received CRT. Moreover, several studies have recently demonstrated that the relevance of the NRF2-lipid peroxidation-ferroptosis axis [40, 41, 47], while another study has reported that the activation of NRF2 signaling alleviates ferroptosis [19].

Ferroptosis, a form of regulated cell death that is different from other forms of cell death, such as apoptosis and necrosis [48], is induced by excessive lipid peroxidation, and it serves as a natural mechanism for tumor inhibition. Targeting ferroptosis represents a promising strategy for cancer treatment, as it helps overcome tumor resistance [29]. The growing recognition of NRF2 as a modulator of ferroptosis indicates that it regulates the sensitivity of cancer treatment in specific types of cancer [49, 50]. For instance, the suppression of NRF2 expression reverses GPX4 inhibitor-induced ferroptosis resistance in head and neck tumors [51] and artesunate-induced ferroptosis resistance in cisplatin-resistant head and neck cancer cells [52]. NRF2 is a major factor that regulates the effects of ferroptosis-related therapies on hepatocellular carcinoma [39]. Erastin, an inducer of ferroptosis, and acetaminophen promote the death of non-small cell lung cancer cells by regulating NRF2 expression [53]. However, their relevance in ESCC radioresistance remains unexplored. Our in vitro data revealed that the overexpression of NRF2 promoted radioresistance in ESCC, largely by inhibiting RT-induced ferroptosis, and the effect of NRF2 overexpression on radioresistance was abrogated by treatment with the ferroptosis inhibitor ferrostatin-1. In recent years, several studies have indicated that ROS-mediated therapeutic resistance is closely related to the regulation of NRF2 expression [54], and ROS-induced lipid peroxidation plays an essential role in ferroptosis [31, 55]. Mechanistically, in the present study, we revealed that the activation of NRF2 weakened radiation-induced ROS generation and lipid peroxidation, alleviating RT-induced ferroptosis and promoting radioresistance in ESCC. We additionally proposed that activated NRF2 could directly combine with the promoter region of SLC7A11 to regulate its transcription, which is related to the inhibition of ferroptosis [56, 57].

SLC7A11, an important component of the cystine/glutamate transporter, regulates cellular lipid peroxidation, and suppresses ferroptosis [58]. Recent studies have demonstrated that SLC7A11 plays diverse functional roles, including the regulation of therapeutic resistance in cancer [38]. Numerous studies have confirmed that therapeutic resistance is associated with SLC7A11 [38]. For instance, the study by Lei et al. demonstrated that the overexpression of SLC7A11 promotes radioresistance, largely via the inhibition of ferroptosis [10]; It has been additionally demonstrated that SLC7A11 is related to radioresistance in human breast cancer and murine melanoma cell lines [59, 60]. Cobler et al. reported that SLC7A11-specific therapeutics would provide tumor-specific radiosensitivity to γ radiation in breast cancer cells, which allows the use of lower radiation doses in treatments, thus producing fewer side effects than other proposed sensitizers [59]. Additionally, previous studies have reported that SLC7A11 may be a potential target gene of NRF2 [42]. For example, Fan et al. [19] mentioned that the activation of NRF2-Keap1 signaling upregulates SLC7A11 and amplifies the secretion of glutamate, thereby affecting the tumor microenvironment [19], while another study demonstrated that the retinal expression of SLC7A11 is regulated by NRF2 in patients with diabetes [61]. The depletion of glutathione in the cortex is associated with the activation of the NRF2 and nuclear factor kappa B signaling pathways, leading to the upregulation of SLC7A11 expression [62]. However, Ono et al. reported that NRF2 does not contribute to the upregulation of SLC7A11 expression through polyunsaturated fatty acids [63]. The results of our IHC analyses revealed that the expression levels of SLC7A11 were consistent with those of NRF2, indicating that there was a significant positive correlation between the expression of SLC7A11 and NRF2. We also observed that the expression of NRF2 substantially increased the expression of SLC7A11 in ESCC cell lines. Interestingly, the results of our dual-luciferase reporter assay and ChIP-sequencing analysis revealed that NRF2 directly binds to the promoter region of SLC7A11 to regulate the expression of SLC7A11. These results support the interaction between NRF2 and SLC7A11 in ESCC and describe their underlying influence on treatment response and prognosis.

As mentioned above, despite the accumulating evidence on the relationship between NRF2 and SLC7A11 expression, in addition to their effect on radioresistance in various types of cancers, the effect of SLC7A11 and NRF2 on radiosensitivity in ESCC remains to be elucidated. We analyzed the correlation between the clinicopathological characteristics, including the expression of SLC7A11 and NRF2 and the efficacy of CRT, and the results demonstrated that higher expression levels of SLC7A11 and NRF2 were related to a poor therapy response in patients with ESCC who received CRT. We additionally investigated whether the inhibition of SLC7A11 expression sensitizes NRF2-overexpressing ESCC tumor cells to RT by inducing ferroptosis. We observed that pre-treatment with SLC7A11-siRNA potently sensitized NRF2 overexpression in vitro. Altogether, our results demonstrate that NRF2 partly mediates RT resistance via the SLC7A11-mediated inhibition of ferroptosis.

Additionally, growing evidence has demonstrated that SLC7A11 is overexpressed in various types of cancers and is associated with patients' poor prognosis [38]. Shiozaki et al. suggested that the expression of SLC7A11 could affect the G1/S checkpoint in ESCC cells as well as the prognosis of patients with ESCC who have undergone esophagectomy [7]. However, the correlation between the expression of SLC7A11 and prognosis of patients with ESCC who received CRT has not been investigated to date. Zhang et al. have previously reported the prognostic value of SLC7A11 in patients with liver cancer [64]. The study by Ma et al. demonstrated that the overexpression of SLC7A11 is a prognostic factor for poor rates of OS and high rates of postoperative recurrence in laryngeal squamous cell carcinoma [21]. In the current study, univariate and multivariate analysis results revealed that the expression of SLC7A11 is an independent prognostic factor for ESCC. We found that high levels of NRF2 or SLC7A11 were associated with low OS, PFS, and poor treatment response in patients with ESCC. Zhong et al. reported that the expression of SLC7A11 promotes metastasis, which is related to a reduced survival rate in patients with prostate cancer [65]. Similarly, we observed that the expression of SLC7A11 was positively correlated with lymph node metastasis, suggesting that SLC7A11 may have a prognostic effect on patients with ESCC via the regulation of lymph node invasion. This indicated that the expression of SLC7A11 might be a potentially useful indicator for selecting irradiated areas of lymphatic drainage. Therefore, SLC7A11 may serve as a potential novel target for cancer therapy and may enhance radiosensitivity.

Taken together, our results provide definitive evidence linking NRF2 with SLC7A11-mediated ferroptosis and elucidate the radioresistance of ESCC. We speculate that a better understanding of the underlying tumor contexts would maximize the efficacy of cancer therapy. NRF2/SLC7A11/ferroptosis-specific therapeutics may provide tumor-specific sensitization to RT in patients with ESCC who develop de novo radioresistance. The exploration of the combination of NRF2/SLC7A11 inhibitors or ferroptosis inducers and radiotherapy remains an important area for future research.

## Conclusions

In summary, the results of this study demonstrated that SLC7A11 plays an important role in the radiosensitivity of ESCC cells by interacting with NRF2, and that expression of SLC7A11 is related to the radiosensitivity and long-term prognosis of ESCC following RT. To the best of our knowledge, the present study is the first to examine the correlation between SLC7A11 and NRF2 and investigate the clinicopathological and prognostic significance of SLC7A11 expression through IHC analysis of ESCC samples from patients who received RT. Immunohistochemical staining showed that the expression of SLC7A11 was an independent prognostic factor in patients with ESCC who received CRT. Furthermore, the study revealed that ESCC radiosensitivity could be potentially improved by targeting the NRF2/SLC7A11/ferroptosis pathway. An greater understanding of the role of SLC7A11 might lead to its use as an important biomarker in ESCC.

## Supplementary Information

**Additional file 1: Figure S1.** Hyperactive NRF2 promotes radioresistance by inhibiting ferroptosis in ESCC cells. (a) Total ROS levels assessment in NC and NF-OE KYSE 30 and KYSE 150 cells at 24 h after exposure to 6 Gy of RT. Bar graph showing RT-induced relative fold changes of cellular total ROS levels per 10000 cells via DCFDA staining in the indicated cells. (b) TEM images of NC and NF-OE KYSE 30 cells 24 h after exposure to 6 Gy of RT. Nu, nucleus; red arrows, mitochondria. Scale bars: 5 µm. (c) Representative images of clonogenic survival assays in NC and NF-OE KYSE 30 or KYSE 150 cells pre-treated with 5 μM ferrostatin-1 or DMSO for 24 h, followed by exposure to 0 Gy, 2 Gy, 4 Gy or 6 Gy of RT, respectively. (d) The quantified clonogenic survival assay in NC and NF-OE KYSE 30 or KYSE 150 cells that were pre-treated with 5 μM ferrostatin-1 or DMSO for 24 h followed by 0 Gy, 2 Gy, 4 Gy or 6 Gy of RT, respectively. Each experiment was conducted independently triplicate. Student’s t-test (two-tailed) was used for statistical analysis, *p < 0.05, **p < 0.01, ***p < 0.001, ****p < 0.0001. Bar graphs: mean ± SEM, n = 3.

**Additional file 2: Figure S2.** Hyperactive NRF2 inhibits RT-induced ferroptosis by regulating SLC7A11 in ESCC cells. (a) Total ROS levels assessment in NF-OE-SLC-Con and NF-OE-SLC-Si KYSE 150 cells at 24 h after exposure to 6 Gy of RT. Bar graph showing RT-induced relative fold changes of cellular total ROS levels per 10000 cells by DCFDA staining in the indicated cells. (b) Representative images of clonogenic survival assays in NF-OE-SLC-Con and NF-OE-SLC-Si KYSE 150 cells pre-treated with 5 μM ferrostatin-1 or DMSO for 24 h, followed by exposure to 0 Gy, 2 Gy, or 6 Gy of RT, respectively. (c) The quantified clonogenic survival assay in NF-OE-SLC-Con and NF-OE-SLC-Si KYSE 150 cells that were pre-treated with 5 μM ferrostatin-1 or DMSO for 24 h followed by 0 Gy, 2 Gy or 6 Gy of RT, respectively. Each experiment was conducted independently triplicate. Student’s t-test (two-tailed) was used for statistical analysis, *p < 0.05, **p < 0.01, ***p < 0.001, ****p < 0.0001. Bar graphs: mean ± SEM, n = 3.

## Data Availability

Data are available from the authors upon reasonable request.

## Abbreviations

SLC7A11: Solute carrier family 7 member 11
NRF2: Nuclear factor erythroid-2
ChIP: Chromatin immunoprecipitation
ESCC: Esophageal squamous cell carcinoma
CRT: Chemoradiotherapy
CT: Chemotherapy
IHC: Immunohistochemistry
OS: Overall survival
PBS: Phosphate buffered solution
PFS: Progression-free survival
PR: Partial response
CR: Complete response
SD: Stable disease
PD: Progressive disease
OD: Optical density
ROS: Reactive oxygen species
RT: Radiotherapy
TEM: Transmission electron microscopy
