# Supplementary figures and images for: SLC7A11 regulated by NRF2 modulates esophageal squamous cell carcinoma radiosensitivity by inhibiting ferroptosis
Source: J Transl Med. 2021 Aug 26;19:367. doi: 10.1186/s12967-021-03042-7 (PMC8393811; doi:10.1186/s12967-021-03042-7)

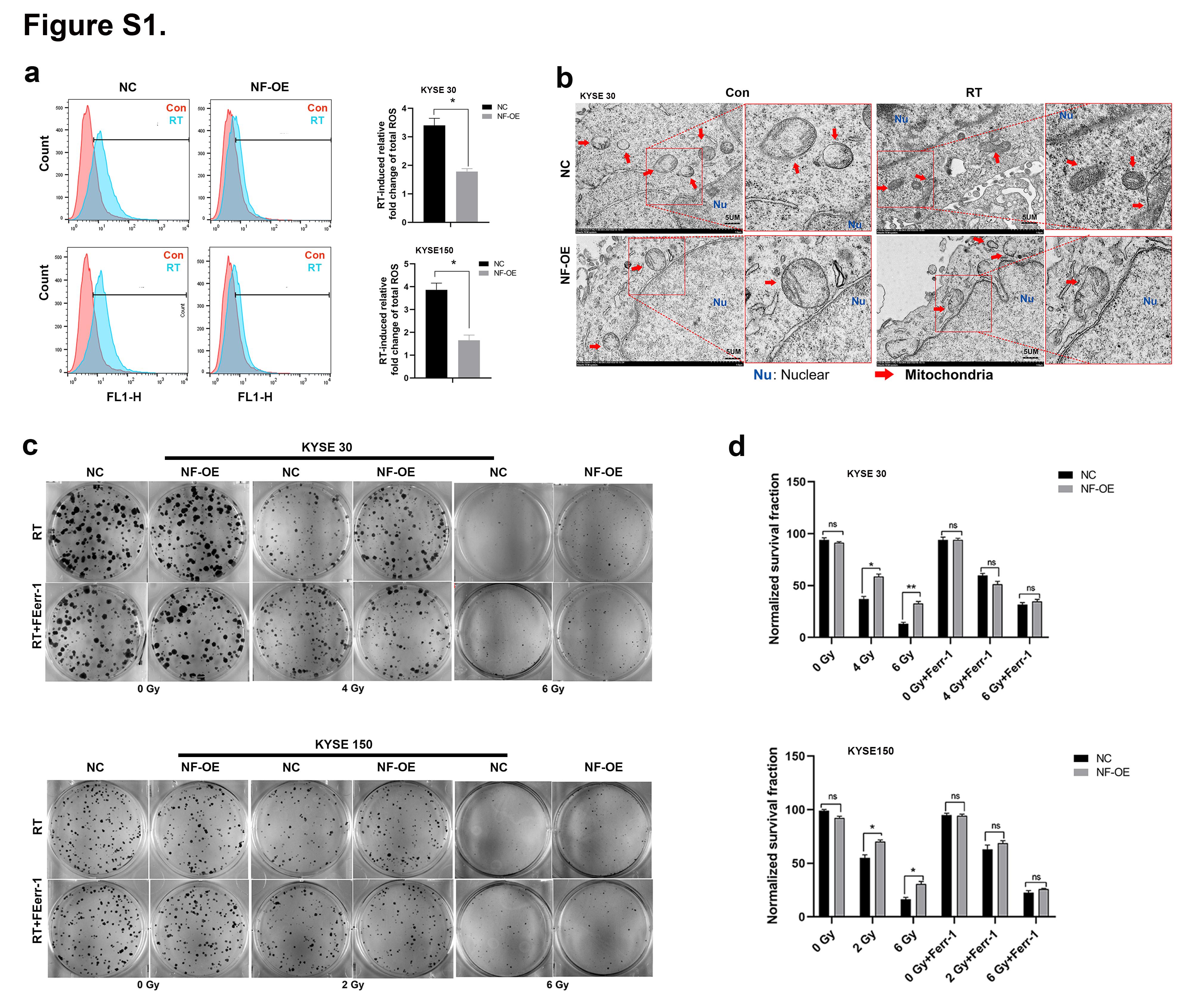

Supplement: Supplementary file 1 — Additional file 1: Figure S1. Hyperactive NRF2 promotes radioresistance by inhibiting ferroptosis in ESCC cells. (a) Total ROS levels assessment in NC and NF-OE KYSE 30 and KYSE 150 cells at 24 h after exposure to 6 Gy of RT. Bar graph showing RT-induced relative fold changes of cellular total ROS levels per 10000 cells via DCFDA staining in the indicated cells. (b) TEM images of NC and NF-OE KYSE 30 cells 24 h after exposure to 6 Gy of RT. Nu, nucleus; red arrows, mitochondria. Scale bars: 5 µm. (c) Representative images of clonogenic survival assays in NC and NF-OE KYSE 30 or KYSE 150 cells pre-treated with 5 μM ferrostatin-1 or DMSO for 24 h, followed by exposure to 0 Gy, 2 Gy, 4 Gy or 6 Gy of RT, respectively. (d) The quantified clonogenic survival assay in NC and NF-OE KYSE 30 or KYSE 150 cells that were pre-treated with 5 μM ferrostatin-1 or DMSO for 24 h followed by 0 Gy, 2 Gy, 4 Gy or 6 Gy of RT, respectively. Each experiment was conducted independently triplicate. Student’s t-test (two-tailed) was used for statistical analysis, *p < 0.05, **p < 0.01, ***p < 0.001, ****p < 0.0001. Bar graphs: mean ± SEM, n = 3. [file 12967_2021_3042_MOESM1_ESM.tif]

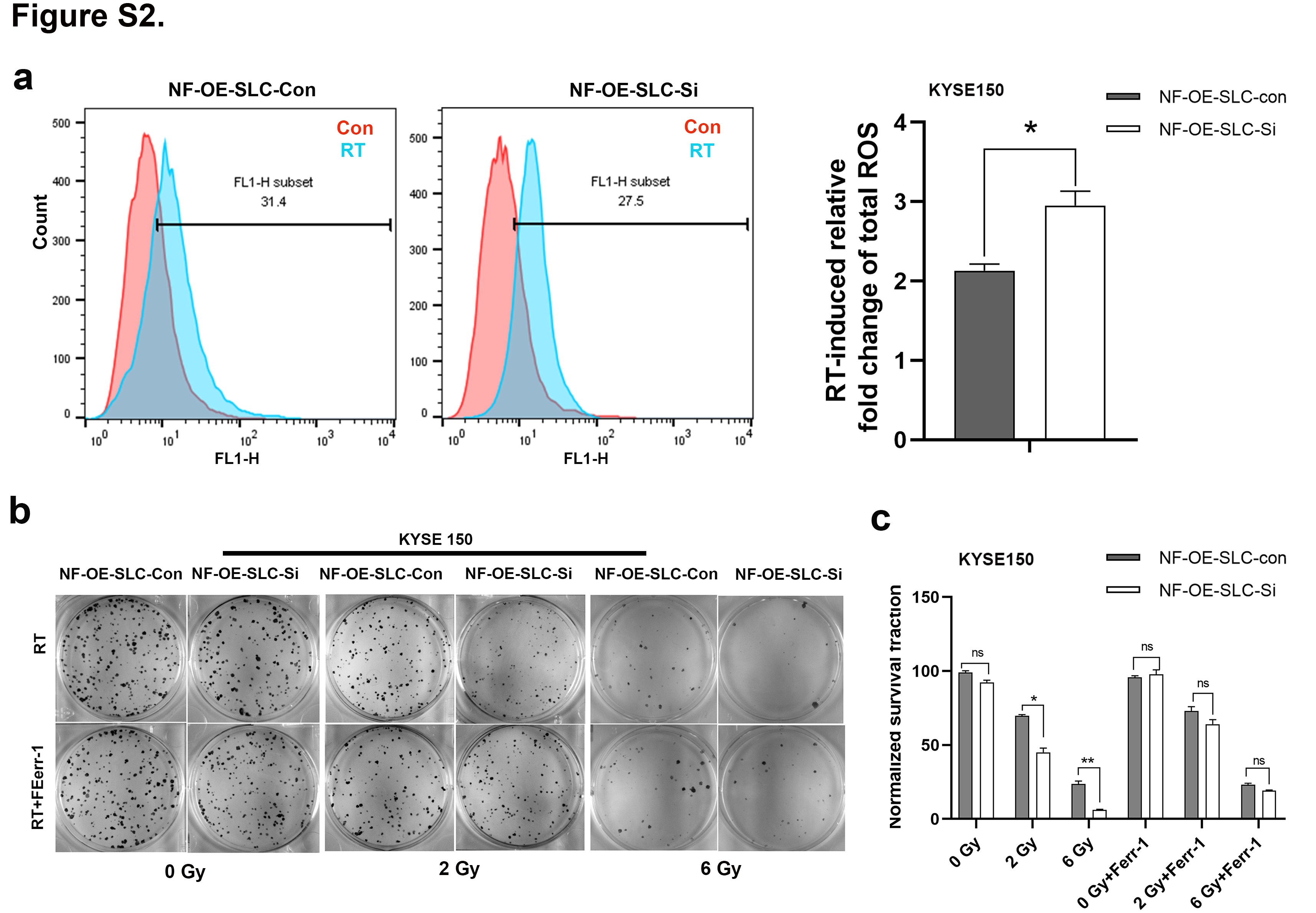

Supplement: Supplementary file 2 — Additional file 2: Figure S2. Hyperactive NRF2 inhibits RT-induced ferroptosis by regulating SLC7A11 in ESCC cells. (a) Total ROS levels assessment in NF-OE-SLC-Con and NF-OE-SLC-Si KYSE 150 cells at 24 h after exposure to 6 Gy of RT. Bar graph showing RT-induced relative fold changes of cellular total ROS levels per 10000 cells by DCFDA staining in the indicated cells. (b) Representative images of clonogenic survival assays in NF-OE-SLC-Con and NF-OE-SLC-Si KYSE 150 cells pre-treated with 5 μM ferrostatin-1 or DMSO for 24 h, followed by exposure to 0 Gy, 2 Gy, or 6 Gy of RT, respectively. (c) The quantified clonogenic survival assay in NF-OE-SLC-Con and NF-OE-SLC-Si KYSE 150 cells that were pre-treated with 5 μM ferrostatin-1 or DMSO for 24 h followed by 0 Gy, 2 Gy or 6 Gy of RT, respectively. Each experiment was conducted independently triplicate. Student’s t-test (two-tailed) was used for statistical analysis, *p < 0.05, **p < 0.01, ***p < 0.001, ****p < 0.0001. Bar graphs: mean ± SEM, n = 3. [file 12967_2021_3042_MOESM2_ESM.tif]
